# Tetrakis(μ-2-chloro­benzoato-κ^2^ *O*:*O*′)bis­[(4-vinyl­pyridine-κ*N*)copper(II)]

**DOI:** 10.1107/S1600536808030766

**Published:** 2008-09-27

**Authors:** Juan Zhao

**Affiliations:** a College of Mechanical Engineering, Qingdao Technological University, Qingdao 266033, People’s Republic of China

## Abstract

The title compound, [Cu_2_(C_7_H_4_ClO_2_)_4_(C_7_H_7_N)_2_], consists of centrosymmetric dinuclear mol­ecules with a Cu⋯Cu separation of 2.6676 (12) Å. In the mol­ecule, four 2-chloro­benzoate anions bridge two Cu^II^ ions, while two neutral 4-vinyl­pyridine ligands coordinate them in axial positions. The Cu^II^ ion has a distorted square-planar pyramidal coordination, with four O atoms from the chlorobenzoate anions at the base. The N pyridine atom completes the coordination environment in the apical position.

## Related literature

In the corresponding dinuclear compound [tetra­kis(μ_2_-acetato)bis­(2-anilinopyridine)dicopper(II)] (Seco *et al.*, 2002 ▶), the Cu^II^ has a distorted square-planar pyramidal coordination environment.
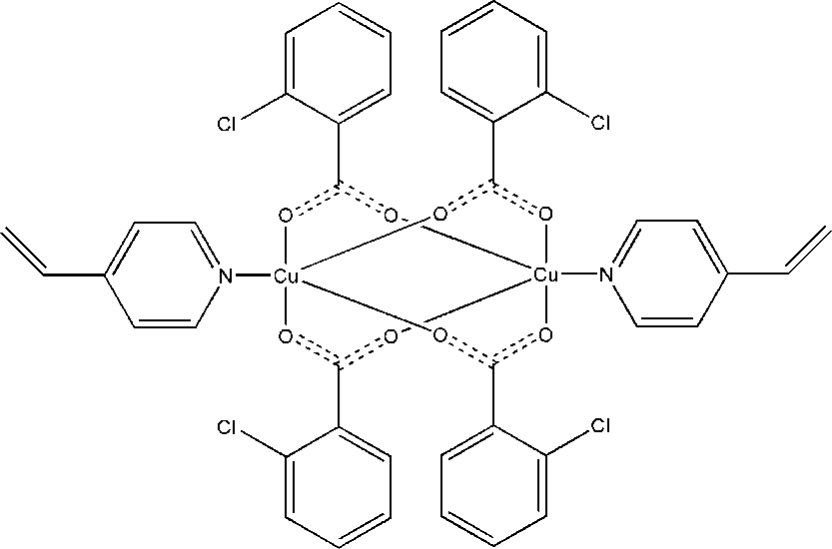


## Experimental

### 

#### Crystal data

[Cu_2_(C_7_H_4_ClO_2_)_4_(C_7_H_7_N)_2_]
*M* *_r_* = 959.58
Monoclinic, 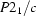

*a* = 10.251 (2) Å
*b* = 20.412 (4) Å
*c* = 10.665 (2) Å
β = 111.99 (3)°
*V* = 2069.2 (8) Å^3^
*Z* = 2
Mo *K*α radiation
μ = 1.34 mm^−1^
*T* = 297 (2) K
0.30 × 0.30 × 0.20 mm

#### Data collection

Enraf–Nonius CAD-4 diffractometer
Absorption correction: ψ scan (North *et al.*, 1968 ▶) *T*_min_ = 0.677, *T*_max_ = 0.767
3708 measured reflections
3689 independent reflections
2587 reflections with *I* > 2σ(*I*)
*R*_int_ = 0.029
3 standard reflections every 100 reflections intensity decay: none

#### Refinement

*R*[*F* ^2^ > 2σ(*F* ^2^)] = 0.059
*wR*(*F* ^2^) = 0.166
*S* = 1.00
3689 reflections
262 parameters
H-atom parameters constrained
Δρ_max_ = 0.37 e Å^−3^
Δρ_min_ = −0.56 e Å^−3^

### 

Data collection: *CAD-4 Software* (Enraf–Nonius, 1989 ▶); cell refinement: *CAD-4 Software*; data reduction: *NRCVAX* (Gabe *et al.*, 1989 ▶); program(s) used to solve structure: *SHELXS97* (Sheldrick, 2008 ▶); program(s) used to refine structure: *SHELXL97* (Sheldrick, 2008 ▶); molecular graphics: *SHELXTL/PC* (Sheldrick, 2008 ▶); software used to prepare material for publication: *WinGX* (Farrugia, 1999 ▶).

## Supplementary Material

Crystal structure: contains datablocks global, I. DOI: 10.1107/S1600536808030766/cv2452sup1.cif

Structure factors: contains datablocks I. DOI: 10.1107/S1600536808030766/cv2452Isup2.hkl

Additional supplementary materials:  crystallographic information; 3D view; checkCIF report

## Figures and Tables

**Fig. 1.**
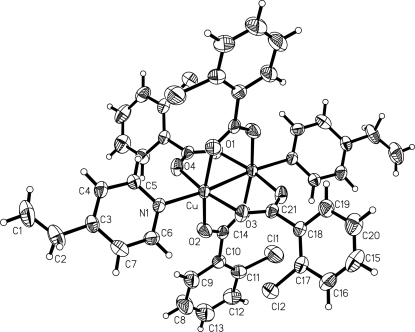
The molecular structure of (I), showing 30% probability displacement ellipsoids and the atom-numbering scheme. Unlabelled atoms are related with the labelled ones by symmetry operation (-x, -y, 3-z).

## supplementary crystallographic information

### Comment

The title compound, (I) (Fig. 1), consists of centrosymmetric dinuclear units, in which four 2-chlorobenzoato groups bridge the two copper ions and a 4-vinylpyridine neutral ligand occupies the axis position of each copper atom, coordinating them through the nitrogen atom. Each copper ion has a distorted square-planar pyramidal coordination, with four oxygen atoms in a plane. The distances for Cu—O1,O2,O3 and O4 are 1.975 (4), 1.957 (4), 1.969 (3) and 1.985 (3) Å, respectively. The fifth coordination position is occupied by the pyridine nitrogen, N, of a ligand molecule at 2.134 (4) Å. All these values agree well with those observed in [Cu_2_(υ-OOCCH_3_)_4_(PhNHpy)_2_] (PhNHpy is 2-anilinopyridine) (Seco *et al.*, 2002). The Cu···Cu separation in (I) is 2.6473 (12) Å.

### Experimental

A solution of 4-vinylpyridine (1.05 g, 10 mmol) in alcohol (10 ml) was added to a solution of CuCl_2_.2H_2_O (1.70 g, 10 mmol) and 2-chlorobebziuc acid (1.56 g, 10 mmol) and KOH (0.56 g, 10 mmol) in alcohol (40 ml). The solution was stirred during 2 h and a precipitate was formed. The blue precipitate was filtered off, washed with alcohol and dried *in vacuo* over CaCO_3_. Blue crystals were obtained from recrystallization in alcohol after a few days.

### Refinement

H atoms were positioned geometrically (C—H = 0.93 Å) and allowed to ride on their parent atoms with *U*_iso_(H) = 1.2*U*_eq_(C).

### Crystal data

**Table:**

[Cu_2_(C_7_H_4_ClO_2_)_4_(C_7_H_7_N)_2_]	*F*(000) = 972
*M**_r_* = 959.58	*D*_x_ = 1.540 Mg m^−^^3^
Monoclinic, *P*2_1_/*c*	Mo *K*α radiation, λ = 0.71073 Å
Hall symbol: -P 2ybc	Cell parameters from 25 reflections
*a* = 10.251 (2) Å	θ = 10–14°
*b* = 20.412 (4) Å	µ = 1.34 mm^−^^1^
*c* = 10.665 (2) Å	*T* = 297 K
β = 111.99 (3)°	Block, blue
*V* = 2069.2 (8) Å^3^	0.30 × 0.30 × 0.20 mm
*Z* = 2	

### Data collection

**Table:**

Enraf–Nonius CAD-4 diffractometer	2587 reflections with *I* > 2σ(*I*)
Radiation source: fine-focus sealed tube	*R*_int_ = 0.029
graphite	θ_max_ = 25.2°, θ_min_ = 2.0°
ω scans	*h* = −12→11
Absorption correction: ψ scan (North et al., 1968)	*k* = 0→24
*T*_min_ = 0.677, *T*_max_ = 0.767	*l* = 0→12
3708 measured reflections	3 standard reflections every 100 reflections
3689 independent reflections	intensity decay: none

### Refinement

**Table:**

Refinement on *F*^2^	Primary atom site location: structure-invariant direct methods
Least-squares matrix: full	Secondary atom site location: difference Fourier map
*R*[*F*^2^ > 2σ(*F*^2^)] = 0.059	Hydrogen site location: inferred from neighbouring sites
*wR*(*F*^2^) = 0.166	H-atom parameters constrained
*S* = 1.00	*w* = 1/[σ^2^(*F*_o_^2^) + (0.1*P*)^2^ + 0.5*P*] where *P* = (*F*_o_^2^ + 2*F*_c_^2^)/3
3689 reflections	(Δ/σ)_max_ < 0.001
262 parameters	Δρ_max_ = 0.37 e Å^−^^3^
0 restraints	Δρ_min_ = −0.56 e Å^−^^3^

### Special details

**Table:**

Geometry. All e.s.d.'s (except the e.s.d. in the dihedral angle between two l.s. planes) are estimated using the full covariance matrix. The cell e.s.d.'s are taken into account individually in the estimation of e.s.d.'s in distances, angles and torsion angles; correlations between e.s.d.'s in cell parameters are only used when they are defined by crystal symmetry. An approximate (isotropic) treatment of cell e.s.d.'s is used for estimating e.s.d.'s involving l.s. planes.
Refinement. Refinement of *F*^2^ against ALL reflections. The weighted *R*-factor *wR* and goodness of fit *S* are based on *F*^2^, conventional *R*-factors *R* are based on *F*, with *F* set to zero for negative *F*^2^. The threshold expression of *F*^2^ > σ(*F*^2^) is used only for calculating *R*-factors(gt) *etc*. and is not relevant to the choice of reflections for refinement. *R*-factors based on *F*^2^ are statistically about twice as large as those based on *F*, and *R*- factors based on ALL data will be even larger.

### Fractional atomic coordinates and isotropic or equivalent isotropic displacement parameters (Å^2^)

**Table:**

	*x*	*y*	*z*	*U*_iso_*/*U*_eq_	
Cu	−0.05229 (6)	0.05461 (3)	1.53164 (6)	0.0465 (2)	
N1	−0.1437 (4)	0.1341 (2)	1.5999 (4)	0.0483 (9)	
Cl1	0.52811 (19)	−0.03630 (12)	1.6765 (3)	0.1233 (9)	
Cl2	0.2716 (2)	0.17779 (10)	1.41176 (18)	0.0975 (6)	
O1	−0.2292 (4)	0.0228 (2)	1.3923 (4)	0.0691 (10)	
O2	0.1417 (4)	0.0684 (2)	1.6572 (4)	0.0707 (11)	
O3	−0.0022 (4)	0.09641 (17)	1.3891 (4)	0.0610 (9)	
O4	−0.0849 (4)	−0.00570 (19)	1.6630 (3)	0.0634 (10)	
C1	−0.4145 (9)	0.2796 (5)	1.8136 (7)	0.119 (3)	
H1A	−0.4628	0.2401	1.8018	0.142*	
H1B	−0.4424	0.3151	1.8522	0.142*	
C2	−0.3097 (7)	0.2851 (3)	1.7770 (5)	0.0787 (18)	
H2A	−0.2638	0.3253	1.7903	0.094*	
C3	−0.2568 (6)	0.2323 (3)	1.7152 (5)	0.0568 (13)	
C4	−0.3184 (6)	0.1719 (3)	1.6807 (5)	0.0604 (13)	
H4A	−0.4007	0.1627	1.6947	0.072*	
C5	−0.2598 (5)	0.1244 (3)	1.6253 (5)	0.0561 (12)	
H5A	−0.3036	0.0837	1.6050	0.067*	
C6	−0.0865 (5)	0.1938 (3)	1.6306 (5)	0.0604 (13)	
H6A	−0.0062	0.2027	1.6126	0.072*	
C7	−0.1379 (6)	0.2424 (3)	1.6864 (5)	0.0693 (15)	
H7A	−0.0924	0.2827	1.7054	0.083*	
C8	0.5064 (9)	0.1132 (5)	1.9717 (8)	0.125 (3)	
H8A	0.5043	0.1444	2.0344	0.151*	
C9	0.3825 (7)	0.0961 (4)	1.8658 (6)	0.094 (2)	
H9A	0.2991	0.1173	1.8564	0.113*	
C10	0.3818 (5)	0.0475 (3)	1.7737 (5)	0.0560 (13)	
C11	0.5093 (6)	0.0209 (3)	1.7889 (6)	0.0678 (15)	
C12	0.6323 (7)	0.0384 (4)	1.8922 (8)	0.095 (2)	
H12A	0.7168	0.0189	1.8996	0.114*	
C13	0.6297 (8)	0.0848 (5)	1.9844 (8)	0.114 (3)	
H13A	0.7122	0.0967	2.0550	0.137*	
C14	0.2409 (5)	0.0288 (3)	1.6714 (5)	0.0540 (12)	
C15	0.1325 (8)	0.1677 (4)	1.0103 (8)	0.091 (2)	
H15A	0.1470	0.1902	0.9408	0.109*	
C16	0.2011 (7)	0.1873 (3)	1.1424 (7)	0.0791 (18)	
H16A	0.2622	0.2229	1.1625	0.095*	
C17	0.1783 (6)	0.1538 (3)	1.2444 (6)	0.0643 (14)	
C18	0.0894 (5)	0.1012 (2)	1.2185 (5)	0.0495 (11)	
C19	0.0226 (6)	0.0818 (3)	1.0849 (5)	0.0713 (15)	
H19A	−0.0374	0.0458	1.0640	0.086*	
C20	0.0454 (7)	0.1163 (4)	0.9819 (6)	0.091 (2)	
H20A	−0.0006	0.1034	0.8924	0.109*	
C21	0.0558 (5)	0.0649 (3)	1.3239 (5)	0.0488 (12)	

### Atomic displacement parameters (Å^2^)

**Table:**

	*U*^11^	*U*^22^	*U*^33^	*U*^12^	*U*^13^	*U*^23^
Cu	0.0445 (3)	0.0547 (4)	0.0436 (3)	0.0030 (3)	0.0201 (2)	−0.0043 (3)
N1	0.045 (2)	0.059 (2)	0.045 (2)	0.0024 (18)	0.0206 (18)	0.0000 (19)
Cl1	0.0601 (10)	0.1388 (18)	0.160 (2)	0.0017 (10)	0.0292 (12)	−0.0630 (16)
Cl2	0.0979 (12)	0.1124 (14)	0.0906 (12)	−0.0378 (11)	0.0451 (10)	−0.0404 (11)
O1	0.049 (2)	0.093 (3)	0.057 (2)	0.000 (2)	0.0117 (17)	−0.010 (2)
O2	0.053 (2)	0.076 (3)	0.075 (3)	0.0039 (19)	0.0150 (19)	−0.024 (2)
O3	0.073 (2)	0.056 (2)	0.069 (2)	0.0110 (18)	0.044 (2)	0.0070 (18)
O4	0.084 (3)	0.064 (2)	0.056 (2)	0.0137 (19)	0.041 (2)	0.0100 (18)
C1	0.120 (7)	0.134 (7)	0.104 (6)	0.044 (6)	0.044 (5)	−0.036 (5)
C2	0.094 (5)	0.082 (4)	0.049 (3)	0.026 (4)	0.014 (3)	−0.011 (3)
C3	0.063 (3)	0.063 (3)	0.040 (3)	0.013 (3)	0.014 (2)	−0.001 (2)
C4	0.056 (3)	0.069 (4)	0.062 (3)	0.008 (3)	0.030 (3)	−0.002 (3)
C5	0.060 (3)	0.052 (3)	0.060 (3)	−0.002 (2)	0.028 (3)	−0.008 (2)
C6	0.056 (3)	0.054 (3)	0.073 (4)	−0.006 (2)	0.026 (3)	−0.004 (3)
C7	0.077 (4)	0.058 (3)	0.063 (4)	−0.002 (3)	0.015 (3)	−0.011 (3)
C8	0.089 (5)	0.182 (10)	0.089 (5)	−0.022 (6)	0.015 (4)	−0.063 (6)
C9	0.061 (4)	0.140 (7)	0.073 (4)	−0.009 (4)	0.015 (3)	−0.037 (4)
C10	0.051 (3)	0.069 (4)	0.045 (3)	−0.005 (2)	0.014 (2)	0.004 (2)
C11	0.058 (3)	0.071 (4)	0.068 (4)	−0.005 (3)	0.017 (3)	0.000 (3)
C12	0.051 (3)	0.128 (6)	0.090 (5)	−0.007 (4)	0.007 (3)	−0.013 (5)
C13	0.075 (5)	0.161 (8)	0.083 (5)	−0.017 (5)	0.002 (4)	−0.030 (5)
C14	0.053 (3)	0.074 (3)	0.040 (3)	−0.007 (3)	0.023 (2)	0.000 (3)
C15	0.081 (5)	0.114 (6)	0.092 (5)	0.017 (4)	0.048 (4)	0.039 (5)
C16	0.081 (4)	0.075 (4)	0.097 (5)	−0.001 (3)	0.051 (4)	0.017 (4)
C17	0.061 (3)	0.074 (4)	0.068 (4)	0.004 (3)	0.037 (3)	0.001 (3)
C18	0.048 (3)	0.056 (3)	0.049 (3)	0.007 (2)	0.023 (2)	0.006 (2)
C19	0.066 (3)	0.096 (4)	0.047 (3)	−0.002 (3)	0.015 (3)	0.002 (3)
C20	0.085 (5)	0.131 (6)	0.051 (4)	0.010 (5)	0.017 (3)	0.021 (4)
C21	0.038 (2)	0.066 (4)	0.043 (3)	0.003 (2)	0.015 (2)	0.004 (2)

### Geometric parameters (Å, °)

**Table:**

Cu—O2	1.958 (4)	C7—H7A	0.9300
Cu—O3	1.971 (3)	C8—C13	1.350 (12)
Cu—O1	1.975 (4)	C8—C9	1.392 (10)
Cu—O4	1.985 (3)	C8—H8A	0.9300
Cu—N1	2.134 (4)	C9—C10	1.393 (8)
Cu—Cu^i^	2.6676 (12)	C9—H9A	0.9300
N1—C5	1.331 (6)	C10—C11	1.368 (8)
N1—C6	1.339 (6)	C10—C14	1.497 (7)
Cl1—C11	1.735 (6)	C11—C12	1.375 (8)
Cl2—C17	1.750 (6)	C12—C13	1.372 (11)
O1—C14^i^	1.236 (6)	C12—H12A	0.9300
O2—C14	1.262 (6)	C13—H13A	0.9300
O3—C21	1.250 (6)	C14—O1^i^	1.236 (6)
O4—C21^i^	1.240 (6)	C15—C20	1.336 (10)
C1—C2	1.279 (10)	C15—C16	1.378 (10)
C1—H1A	0.9300	C15—H15A	0.9300
C1—H1B	0.9300	C16—C17	1.377 (8)
C2—C3	1.469 (7)	C16—H16A	0.9300
C2—H2A	0.9300	C17—C18	1.369 (7)
C3—C4	1.372 (8)	C18—C19	1.388 (7)
C3—C7	1.379 (8)	C18—C21	1.489 (6)
C4—C5	1.383 (7)	C19—C20	1.397 (8)
C4—H4A	0.9300	C19—H19A	0.9300
C5—H5A	0.9300	C20—H20A	0.9300
C6—C7	1.359 (7)	C21—O4^i^	1.240 (6)
C6—H6A	0.9300		
			
O2—Cu—O3	88.52 (17)	C13—C8—C9	120.6 (8)
O2—Cu—O1	166.73 (16)	C13—C8—H8A	119.7
O3—Cu—O1	89.60 (16)	C9—C8—H8A	119.7
O2—Cu—O4	90.15 (17)	C8—C9—C10	120.9 (7)
O3—Cu—O4	166.87 (14)	C8—C9—H9A	119.6
O1—Cu—O4	88.70 (17)	C10—C9—H9A	119.6
O2—Cu—N1	96.94 (15)	C11—C10—C9	116.5 (5)
O3—Cu—N1	102.02 (14)	C11—C10—C14	127.1 (5)
O1—Cu—N1	96.30 (16)	C9—C10—C14	116.4 (5)
O4—Cu—N1	91.12 (15)	C10—C11—C12	122.7 (6)
O2—Cu—Cu^i^	83.76 (11)	C10—C11—Cl1	122.3 (4)
O3—Cu—Cu^i^	85.49 (10)	C12—C11—Cl1	115.0 (5)
O1—Cu—Cu^i^	83.00 (12)	C13—C12—C11	119.7 (7)
O4—Cu—Cu^i^	81.38 (11)	C13—C12—H12A	120.1
N1—Cu—Cu^i^	172.47 (11)	C11—C12—H12A	120.1
C5—N1—C6	115.5 (4)	C8—C13—C12	119.5 (7)
C5—N1—Cu	119.7 (3)	C8—C13—H13A	120.2
C6—N1—Cu	124.6 (3)	C12—C13—H13A	120.2
C14^i^—O1—Cu	124.5 (4)	O1^i^—C14—O2	124.9 (5)
C14—O2—Cu	123.8 (4)	O1^i^—C14—C10	119.1 (5)
C21—O3—Cu	121.5 (3)	O2—C14—C10	116.0 (5)
C21^i^—O4—Cu	126.0 (3)	C20—C15—C16	120.2 (6)
C2—C1—H1A	120.0	C20—C15—H15A	119.9
C2—C1—H1B	120.0	C16—C15—H15A	119.9
H1A—C1—H1B	120.0	C17—C16—C15	119.4 (6)
C1—C2—C3	124.5 (8)	C17—C16—H16A	120.3
C1—C2—H2A	117.8	C15—C16—H16A	120.3
C3—C2—H2A	117.8	C18—C17—C16	121.9 (6)
C4—C3—C7	115.5 (5)	C18—C17—Cl2	119.6 (4)
C4—C3—C2	124.8 (5)	C16—C17—Cl2	118.5 (5)
C7—C3—C2	119.7 (6)	C17—C18—C19	117.8 (5)
C3—C4—C5	121.0 (5)	C17—C18—C21	124.3 (5)
C3—C4—H4A	119.5	C19—C18—C21	117.9 (5)
C5—C4—H4A	119.5	C18—C19—C20	120.0 (6)
N1—C5—C4	123.0 (5)	C18—C19—H19A	120.0
N1—C5—H5A	118.5	C20—C19—H19A	120.0
C4—C5—H5A	118.5	C15—C20—C19	120.8 (7)
N1—C6—C7	124.4 (5)	C15—C20—H20A	119.6
N1—C6—H6A	117.8	C19—C20—H20A	119.6
C7—C6—H6A	117.8	O4^i^—C21—O3	125.6 (4)
C6—C7—C3	120.5 (5)	O4^i^—C21—C18	117.1 (4)
C6—C7—H7A	119.7	O3—C21—C18	117.2 (4)
C3—C7—H7A	119.7		
			
O2—Cu—N1—C5	−132.2 (4)	C4—C3—C7—C6	−1.5 (8)
O3—Cu—N1—C5	137.8 (4)	C2—C3—C7—C6	179.3 (5)
O1—Cu—N1—C5	46.9 (4)	C13—C8—C9—C10	2.6 (15)
O4—Cu—N1—C5	−41.9 (4)	C8—C9—C10—C11	−3.5 (11)
O3—Cu—N1—C6	−47.9 (4)	C8—C9—C10—C14	174.7 (7)
O1—Cu—N1—C6	−138.8 (4)	C9—C10—C11—C12	2.6 (9)
O4—Cu—N1—C6	132.4 (4)	C14—C10—C11—C12	−175.4 (6)
O2—Cu—O1—C14^i^	4.6 (10)	C9—C10—C11—Cl1	−175.9 (5)
O3—Cu—O1—C14^i^	86.4 (4)	C14—C10—C11—Cl1	6.1 (8)
O4—Cu—O1—C14^i^	−80.5 (4)	C10—C11—C12—C13	−0.5 (12)
N1—Cu—O1—C14^i^	−171.5 (4)	Cl1—C11—C12—C13	178.0 (7)
Cu^i^—Cu—O1—C14^i^	0.9 (4)	C9—C8—C13—C12	−0.4 (16)
O3—Cu—O2—C14	−86.8 (4)	C11—C12—C13—C8	−0.6 (14)
O1—Cu—O2—C14	−4.8 (10)	Cu—O2—C14—O1^i^	0.8 (8)
O4—Cu—O2—C14	80.2 (4)	Cu—O2—C14—C10	−179.3 (3)
N1—Cu—O2—C14	171.3 (4)	C11—C10—C14—O1^i^	14.1 (8)
Cu^i^—Cu—O2—C14	−1.1 (4)	C9—C10—C14—O1^i^	−163.9 (6)
O2—Cu—O3—C21	82.9 (4)	C11—C10—C14—O2	−165.8 (6)
O1—Cu—O3—C21	−84.0 (4)	C9—C10—C14—O2	16.2 (7)
O4—Cu—O3—C21	−1.4 (9)	C20—C15—C16—C17	−0.3 (11)
N1—Cu—O3—C21	179.7 (4)	C15—C16—C17—C18	0.1 (9)
Cu^i^—Cu—O3—C21	−1.0 (4)	C15—C16—C17—Cl2	177.6 (5)
O2—Cu—O4—C21^i^	−84.4 (4)	C16—C17—C18—C19	0.6 (8)
O3—Cu—O4—C21^i^	−0.3 (10)	Cl2—C17—C18—C19	−176.9 (4)
O1—Cu—O4—C21^i^	82.4 (4)	C16—C17—C18—C21	−177.2 (5)
N1—Cu—O4—C21^i^	178.6 (4)	Cl2—C17—C18—C21	5.3 (7)
Cu^i^—Cu—O4—C21^i^	−0.8 (4)	C17—C18—C19—C20	−1.0 (8)
C1—C2—C3—C4	4.6 (10)	C21—C18—C19—C20	176.9 (5)
C1—C2—C3—C7	−176.3 (7)	C16—C15—C20—C19	−0.1 (11)
C7—C3—C4—C5	2.2 (8)	C18—C19—C20—C15	0.8 (10)
C2—C3—C4—C5	−178.6 (5)	Cu—O3—C21—O4^i^	1.9 (7)
C6—N1—C5—C4	−0.5 (7)	Cu—O3—C21—C18	−179.9 (3)
Cu—N1—C5—C4	174.3 (4)	C17—C18—C21—O4^i^	−120.3 (6)
C3—C4—C5—N1	−1.3 (8)	C19—C18—C21—O4^i^	61.9 (6)
C5—N1—C6—C7	1.2 (8)	C17—C18—C21—O3	61.3 (7)
Cu—N1—C6—C7	−173.3 (4)	C19—C18—C21—O3	−116.5 (6)
N1—C6—C7—C3	−0.2 (9)		

Symmetry codes: (i) −*x*, −*y*, −*z*+3.
